# Automated Laryngeal Cancer Detection and Classification Using Dwarf Mongoose Optimization Algorithm with Deep Learning

**DOI:** 10.3390/cancers16010181

**Published:** 2023-12-29

**Authors:** Nuzaiha Mohamed, Reem Lafi Almutairi, Sayda Abdelrahim, Randa Alharbi, Fahad Mohammed Alhomayani, Bushra M. Elamin Elnaim, Azhari A. Elhag, Rajendra Dhakal

**Affiliations:** 1 Department of Public Health, College of Public Health and Health Informatics, University of Hail, Ha’il 81451, Saudi Arabia; noi.idrs@uoh.edu.sa (N.M.); r.almutairi@uoh.edu.sa (R.L.A.); soa.abdalraheem@uoh.edu.sa (S.A.); 2 Department of Statistics, Faculty of Science, University of Tabuk, Tabuk 71491, Saudi Arabia; ralharbi@ut.edu.sa; 3 College of Computers and Information Technology, Taif University, Taif 21944, Saudi Arabia; fahad@tu.edu.sa; 4 Applied College, Taif University, Taif 21944, Saudi Arabia; 5 Department of Computer Science, College of Science and Humanities in Al-Sulail, Prince Sattam Bin Abdulaziz University, Al-Kharj 16278, Saudi Arabia; b.elamin@psau.edu.sa; 6 Department of Mathematics and Statistics, College of Science, Taif University, Taif 21944, Saudi Arabia; a.alhag@tu.edu.sa; 7 Department of Computer Science and Engineering, Sejong University, Seoul 05006, Republic of Korea

**Keywords:** laryngeal cancer, Dwarf Mongoose Optimization, deep learning, endoscopy, median filtering, multi-head bidirectional gated recurrent unit

## Abstract

**Simple Summary:**

Laryngeal cancer poses a major global health burden, with late-stage diagnoses contributing to decreased survival rates. Recently, deep learning and deep convolutional neural network models have exhibited significant attention in the diagnosis of various diseases like skin cancer and diabetic retinopathy. Therefore, this study focuses on the design and development of a deep learning-based laryngeal cancer detection and classification model. The proposed model exploited a hyperparameter-tuned EfficientNetB0 model with a multi-head bidirectional gated recurrent unit for classification. In addition, the Dwarf Mongoose Optimization algorithm is applied for the hyperparameter tuning process. The experimental results stated that the proposed model is found to be an accurate and reliable approach for automated detection of laryngeal cancer.

**Abstract:**

Laryngeal cancer (LCA) is a serious disease with a concerning global rise in incidence. Accurate treatment for LCA is particularly challenging in later stages, due to its complex nature as a head and neck malignancy. To address this challenge, researchers have been actively developing various analysis methods and tools to assist medical professionals in efficient LCA identification. However, existing tools and methods often suffer from various limitations, including low accuracy in early-stage LCA detection, high computational complexity, and lengthy patient screening times. With this motivation, this study presents an Automated Laryngeal Cancer Detection and Classification using a Dwarf Mongoose Optimization Algorithm with Deep Learning (ALCAD-DMODL) technique. The main objective of the ALCAD-DMODL method is to recognize the existence of LCA using the DL model. In the presented ALCAD-DMODL technique, a median filtering (MF)-based noise removal process takes place to get rid of the noise. Additionally, the ALCAD-DMODL technique involves the EfficientNet-B0 model for deriving feature vectors from the pre-processed images. For optimal hyperparameter tuning of the EfficientNet-B0 model, the DMO algorithm can be applied to select the parameters. Finally, the multi-head bidirectional gated recurrent unit (MBGRU) model is applied for the recognition and classification of LCA. The simulation result analysis of the ALCAD-DMODL technique is carried out on the throat region image dataset. The comparison study stated the supremacy of the ALCAD-DMODL technique in terms of distinct measures.

## 1. Introduction

Laryngeal cancer (LCA) is one of the major and preeminent malignant tumors of the neck and head area. Treatment results of LCA in an earlier phase are good, whereby five-year patient survival rates with Tis, T1, and T2 LCA range around 80–90% [1]. While endoscopy becomes the major tool for identifying LCA in medical applications, endoscopy with standard white light can be confined for both contrast and resolution which provides the management or misdiagnosis of superficial mucosal cancer and the pioneering lesions associated with it, still by expert endoscopists. In contrast, unwanted biopsy and suspicious cancer identification are the second main difficulties in medical practices because of an intrinsic concern of endoscopists to prevent the onset of early-stage cancer. Consequently, the majority of the patients acquire their diagnoses at the final phase as well as frequently endure vocal function loss impacting the deterioration of life quality. [2]. In recent times, endoscopic techniques with narrow-band imaging (NBI) that increase the analysis of epithelial and sub-epithelial microvascular patterns played a crucial part in earlier recognition of LCA [3]. However, the usage of NBI for diagnoses needs innovative magnifying endoscopes, a particular training period, and practiced endoscopists that confine the medical utilization of NBI endoscopy in numerous emerging nations like China [4]. Hence, the utilization of conventional non-magnifying and white-light images for LCA analysis is not only significant but also essential for less-developed countries or regions facing challenges such as a shortage of skilled endoscopists and a lack of advanced imaging endoscopes [5].

Because of the specific physiological features and structures, it is normally complicated for human eyes to capture irrelevant LCA lesions from non-magnified endoscopy [6]. Furthermore, as machine learning (ML) methods develop quickly, intelligent and accurate diagnoses have the potential with image-based deep learning (DL) [7]. Now, DL states to an ML method that is dependent upon a neural network (NN) model with numerous data representation stages. Convolutional neural networks (CNNs) constitute feedforward neural networks (FFNNs) with deep architecture and convolution computation [8]. It has a model that must overcome classification and identification issues. By comparison with standard image processing techniques, CNN has a higher ability for evaluation and feature extraction [9]. Presently, artificial intelligence (AI) depends on deep CNNs (DCNNs) that could be implemented in pathology, magnetic resonance images (MRIs), classification of skin cancer, congenital cataracts, and diabetic retinopathy (DR) analysis [10]. With the help of such cutting-edge DL methods, the AI technique promptly offered accurate analyses depending on image data that must be possibly provided for identifying early diseases as well as improving the survival rate of patients.

This study presents an Automated Laryngeal Cancer Detection and Classification using a Dwarf Mongoose Optimization Algorithm with Deep Learning (ALCAD-DMODL) technique. The main aim of the ALCAD-DMODL method is to recognize the existence of LCA using the DL model. In the presented ALCAD-DMODL technique, a median filtering (MF)-based noise removal process takes place to get rid of the noise. Besides, the ALCAD-DMODL technique involves the EfficientNet-B0 model for deriving feature vectors from the pre-processed images. For optimal hyperparameter tuning of the EfficientNet-B0 model, the DMO algorithm can be applied to select the parameters. Finally, the multi-head bidirectional gated recurrent unit (MBGRU) model is applied for the recognition and classification of LCA. The simulation result analysis of the ALCAD-DMODL technique is carried out on the throat region image dataset.

## 2. Literature Works

Alrowais et al. [11] developed an innovative LCA Detection and Classification using the Aquila Optimizer Algorithm with DL (LCDC-AOADL) method. The Inceptionv3 architecture was employed for feature extraction. Additionally, the algorithm implemented a deep belief network (DBN) framework for identifying and classifying LCA. In addition, the AOA should be applied for the hyperparameter tuning of the DBN method which leads to an increase in the detection rate. Zhou et al. [12] presented an LCA classification network (LPCANet) that depends on a CNN and attention module. Initially, the novel HIs have been sequentially collected into patches. Next, the images could be provided input into the simple ResNet-50 for feature extraction. Similarly, position and channel attention mechanisms can be included as equivalent. Also, the fusion feature map was removed as well as visually evaluated by the Grad_CAM to offer a specific explainability for the last outcomes.

In Meyer-Veit et al. [13], an effective HIs-DL technique was projected for predicting LCA. Primarily, an important wavelength analysis was accomplished for identifying the highly useful channels in the HS cubes for decreasing the noise as well as increasing the prediction. According to the outcomes, a new Unet, named the EFX-Unet, has been designed as well as two channels in all cubes that could be employed for prediction and training. You et al. [14] projected consistent estimates of present DL methods. This research generated white-light and NBI image databases of vocal cord leukoplakia that can be categorized into six types. Vocal cord leukoplakia classification could be executed by six traditional DL techniques, namely Vision Transformer, AlexNet, DenseNet, VGG, ResNet, and Google Inception. DenseNet-121, ResNet-152, and GoogLeNet carried out exceptional classification.

Ayyaz et al. [15] considered a novel hybrid technique that includes seven important stages. This method can choose two various CNN techniques (Alexnet and VGG19) for removing features. The transfer learning (TL) algorithms have been implemented. The approach also employed a genetic algorithm (GA) in FS. This method also combined the chosen features of two architectures through a serial-based technique. Lastly, the preeminent features have contributed to numerous ML methods for classification and detection. In Kwon et al. [16], DL-based CNN methods have been developed and categorized using LCA images and voice data. Accurate classification might be acquired by implementing decision tree (DT) ensemble learning employing the possibility of the CNN classifier method. The classification and regression tree (CART) technique could be implemented. Next, the authors related the classification precision of DT ensemble learning with CNN separate classification by combining the laryngeal image with the voice DT algorithm.

In Lubrano et al. [17], the authors examined the capability of DL to support the pathologist with automatic and dependable categorization of HI lesions. A huge dataset of HIs (>2000 slides) is planned for emerging as an automatic analytical tool. This introduced analysis also designed and trained an uncertainly supervised method executing classification in whole-slide images (WSIs). In Huang et al. [18], an end-wise ViT-AMC network (ViT-AMCNet) with adaptive model fusion and multi-objective optimizer to be incorporated as well as to combine the ViT and AMC blocks was designed. Initially, this study evidences the possibility of combining the ViT and AMC blocks dependent upon Hoeffding’s dissimilarity. Afterward, a multi-objective optimizer technique was developed to resolve the issue, in which ViT and AMC blocks do not concurrently provide a better feature representation. Besides, a modified model fusion algorithm combining the fusion and metrics blocks was designed.

## 3. The Proposed Method

**Figure 1:**
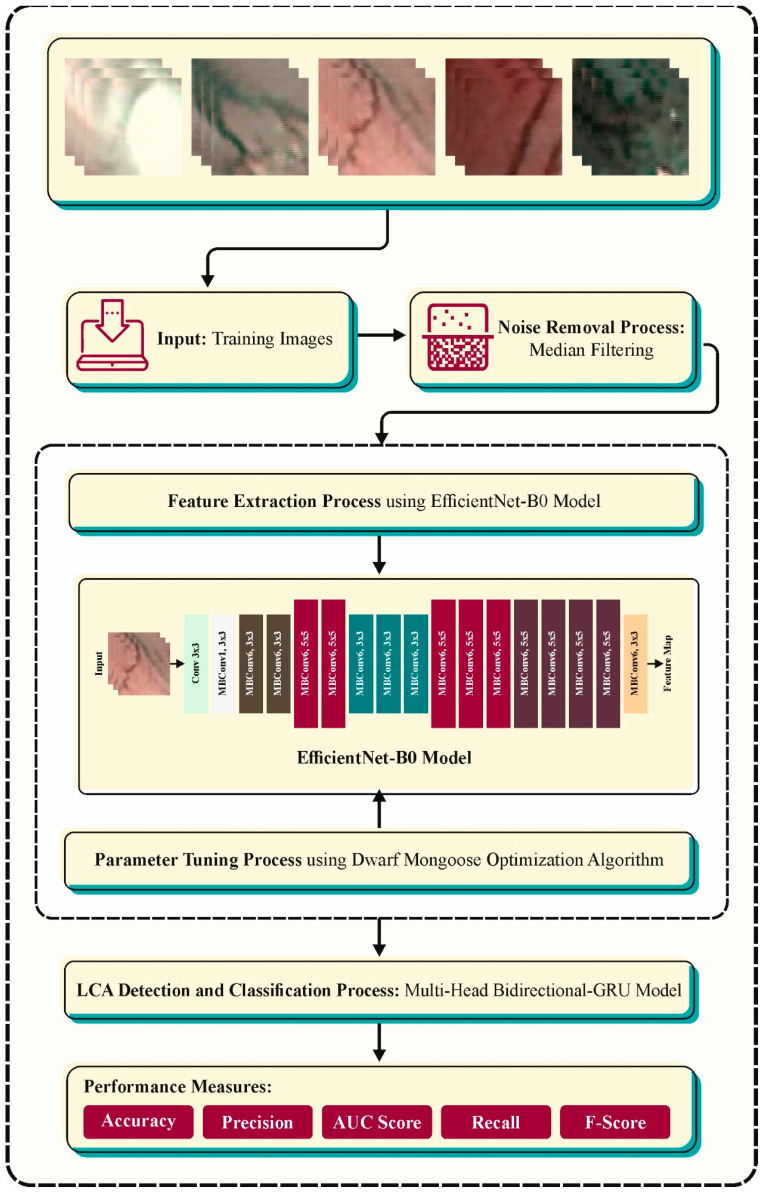
The overall flow of the ALCAD-DMODL technique.

In this study, we have presented an ALCAD-DMODL technique. The main aim of the ALCAD-DMODL system is to recognize the existence of LCA using the DL model. The presented ALCAD-DMODL technique comprises MF-based preprocessing, an EfficientNet-B0-based feature extractor, DMO-based parameter tuning, and MBGRU-based classification. Figure 1 illustrates the entire flow of the ALCAD-DMODL algorithm. The figure shows that the ALCAD-DMODL technique, derived from automated laryngeal cancer recognition and classification, operates by meticulous, multiple-step processes. This procedure starts with tackling unwanted noise within the throat region images. An MF approach effectively removes noise but maintains vital image details, making sure there is reliable information for subsequent phases. During this work, the EfficientNet-B0, a pre-trained DL approach, has been trained on a huge database of images. This robust model examines the pre-processed images and extracts useful feature vectors, basically condensed representations of the main features in all the images.

These vectors capture key data on the throat area, paving the way for correct cancer recognition. EfficientNet-B0 depends on distinct internal parameters that greatly influence its solution. At this point, the DMO approach comes into play. Simulated by the co-operative hunting behavior of dwarf mongooses, DMO wisely searches for the boosting integration of these parameters, adjusting EfficientNet-B0 for peak accuracy in laryngeal cancer recognition. Eventually, the extracting feature vectors and optimizer EfficientNet-B0 approach provide data to the powerful MBGRU network. This advanced recurrent neural network (RNN), planned to procedure sequential data such as image series, examines the features and outcomes of a definitive classification, namely cancerous and non-cancerous.

### 3.1. Preprocessing 

Primarily, the MF-based noise removal process takes place to get rid of the noise. MF has deployed image pre-processing methods to assist in mitigating noise and improving the digital image qualities [19].

In the context of medical image or computer vision (CV) tasks, namely LCA recognition, this technique is particularly valued. The basic principle of MF includes exchanging all the pixel’s intensity values with the median value of its adjacent pixels. A different mean filter that assumes the average intensity, MF is robust to outliers, making it effective in maintaining image edges and fine details but efficiently suppressing salt-and-pepper or random noise. This characteristic is essential in enhancing the entire clarity of throat area images, permitting later DL approaches to focus on significant features for reliable and accurate LCA recognition. Combining MF as part of the pre-processing pipeline gives a further resilient and noise-resistant input, finally improving the solution of the following analytical stages.

### 3.2. EfficientNet-B0 Model

At this stage, the ALCAD-DMODL technique involves the EfficientNet-B0 model for deriving feature vectors from the pre-processed images. The EfficientNet family of structures is established to determine a suitable process to measure CNNs and enhance network solutions [20]. This study developed a compound scaling method that consistently scales depth, width, and resolution utilizing a provided group of coefficients. With the help of such a process, the authors are capable of making the Efficientnet-B0-CNNs structure. The EfficientNet technique set contains 8 approaches from B0 to B7, all the subsequent model counts mentioning variations with additional parameters and maximum accurateness.

CNNs capture richer and more difficult features by fine-tuning the network depth. However, the vanishing gradient issue creates very complex network training. The model has been gathering further fine-grained features by altering its width. Training can easily depict the detailed baseline EfficientNetB0 technique that takes 224×224×3 input images, but 224×224 has the image’s width and height and 3 represents the image’s dimension. This method utilized several convolution layers with a 3×3 receptive region and the mobile reversed bottleneck convolution for capturing features across layers.

(1) $$w=\beta^{\phi },$$

(2) $$d=\alpha^{\phi },$$

(3) $$r=\gamma^{\phi },$$

(4) $$s.t\;\alpha .\beta^{2}.\gamma^{2}\approx 2$$

(5) α≥1,β≥1,γ≥1.

whereas w refers to the width, d implies the height, and r signifies the resolution, α, β, and γ denote the constant coefficients defined by a smaller grid search. Depth, width, and resolution of the network can be uniformly computed by EfficientNet utilizing a multiple co-efficient Φ.

Conversely, extensive and shallow networks can be impotent in obtaining higher-level features. Higher-resolution images allow CNNs to identify additional time patterns, and further memory and processing power are required to perform greater images. Additionally, EfficientNet is mostly suitable to employ DL on edge, while it decreases computational rate, battery utility, and training and implication speeds. The type of architecture performance finally allows the utilization of DL with mobile and other edge devices.

### 3.3. DMO-Based Hyperparameter Tuning

For optimal hyperparameter tuning of the EfficientNet-B0 model, the DMO algorithm can be applied to select the parameters. DMO has a new population-based meta-heuristic model that depends on the social and foraging behavior of a dwarf mongoose called Helogale [21]. All separately seek food because the food search cannot be a cooperative practice, then foraging could be mutually achieved because of the seminomadic features of such animals, the structure of the sleeping mound (SM) was nearer to a relevant food source. The mathematical models are used for resolving optimizer problems.

The method initiates with random initialization. Subsequently, all decisions are collected in the global preeminent optimum owing to the diversification and intensification procedures. Similarly, the DMO activates its result by modifying the DMO population. It could be randomly created among the upper and lower limits of the problems.

(6) $$X=\left[\begin{array}{lllll} x_{1,1} & x_{1,2} & \ldots & x_{1,d-1} & x_{1,d}\\ x_{2,1} & x_{2,2} & \ldots & x_{2,d-1} & x_{2,d}\\ & \;\;\vdots \vdots & x_{i,j} & \;\;\;\vdots \vdots & \\ x_{n,1} & x_{n,2} & \ldots & x_{n,d-1} & x_{n,d} \end{array}\right]$$

In Equation (6), $x_{i,j}$ signifies the position of the $j^{th}$ parameter of the $i^{th}$ population, X represents the group of candidates’ present population to be arbitrarily produced, d characterizes the dimensionality of the problem, and n indicates the population size.

(7) $$x_{i,j}=unifrnd\left(VarMin,\;VarMax,\;VarSize\right)$$

where Equation (7), a consistently distributed random number denoted as unifrnd, VarMin and VarMax represents the lower and upper limits. The dimensionality refers to VarSize. The best solution in some rounds will be the over-fit solution.

Comparable to other meta-heuristic methods, there are 2 various phases in the DMO: exploration (a stochastic search for novel SM or food source) or diversification and exploitation (individual mongoose performs a wide-ranging search within the search range), named intensification. The babysitters, alpha, and scout groups are the three social models of the DMO that execute the tasks of the 2 previously mentioned phases.

The family unit controller represents the alpha female (α) and can be designated by the given formula:

(8) $$\alpha =\frac{it_{i}}{\sum_{i=1}^{n}fit_{i}}$$

In Equation (8), peep describes the sound of α, and the no. of mongooses in the alpha group has n−bs, and bs denotes the no. of babysitters.

The SM could be described by the rich food as follows,

(9) $$X_{i+1}=X_{i}+phi\times peep$$

In Equation (9), uniform distribution random number $\left[-1,1\right]$ is phi.

(10) $$sm_{i}=\frac{fit_{i+1}-fit_{i}}{max\left\{\left|fit_{i+1},fit_{i}\right|\right\}}$$

Once an SM determines an average value to be expressed as given below:

(11) $$\varphi =\frac{\sum_{i=1}^{n}sm_{i}}{n}$$

The scouting stands the following stage, while the babysitter alters the rule that evaluates the following SM defined via other food sources.

The scout group drives the search for the following SM to give the exploration because a mongoose can be called not for returning at a prior SM. Concurrently, it is named as scout and forage in DMO.

(12) $$X_{i+1}=\left\{\begin{array}{l} X_{i}-CF\times phi\times rand\times \left[X_{i}-\vec{M}\right]\;if\;\varphi_{i+1}>\varphi_{j}\\ X_{i}+CF\times phi\times rand\times \left[X_{i}-\vec{M}\right]\;else \end{array}\right.$$

In Equation (12), rand is a random integer with [0,1], $CF={\left(1-\frac{iter}{{Max}_{iter}}\right)}^{\left(2\frac{iter}{{Max}_{iter}}\right)}$ represents the parameter for the collective volatile measure of individual movement, which linearly dropped at some iterations. $\vec{M}=\sum_{i=1}^{n}\frac{X_{i}\times sm_{i}}{X_{i}}$ shows the vector to inspire the individual movement for the original SM.

While the foraging and scouting set searches for food sources and SM, the babysitter’s set stands with the children. The no. of members has separated at the whole number of candidate population as not scout or forage till the changes of the babysitter’s parameter are occurred.

The DMO algorithm develops a fitness function (FF) to accomplish a greater classifier solution. It explains positive integers to refer to the best result of candidate efficiency. The decrease in classifier rate of errors has been assumed that FF is written as:

(13) $$fitness\left(x_{i}\right)=ClassifierErrorRate\left(x_{i}\right)\;=\frac{No.\;of\;misclassified\;instances}{Total\;no.\;of\;instances}\times 100$$

### 3.4. Classification Using MBGRU

Finally, the MBGRU architecture has been applied to recognizing and classifying LCA. Different from typical NNs, MBGRU excels at capturing long-range dependencies in sequential data. This performed admirably for LCA recognition, but it efficiently analyzes connections among various areas of the throat images and identifies subtle patterns that can signal cancer development. MBGRU’s several heads permit it to concentrate on distinct features of the input features, extracting more detailed data and potentially leading to optimum model efficiency. By processing the input sequence in either forward or backward directions, MBGRU attains a deeper understanding of feature connections, leading to more robust and accurate classification. The MBGRU receives its input from the EfficientNet-B0 approach—the feature vector extraction from the pre-processed throat area images. These vectors capture the vital features of the images, generating the basis for cancer recognition. After processing the input feature vectors, the MBGRU creates a last classification outcome, signifying if the image depicts signs of laryngeal cancer or not. This result serves as the analysis for the patient. The MBGRU itself has hyperparameters that need careful optimizers for achieving better solutions. These hyperparameters, like the count of hidden units and layers, are tuned utilizing approaches like the DMO system.

RNNs can procedure sequential data [22]. In addition, RNNs are capable of learning any data in preceding data once managing the present data. The LSTM and GRU are enhanced RNN approaches that have potent modeling abilities for extended dependencies, and GRU can reduce difficulties related to LSTM. A GRU has been collected of updated gate $z_{t}$ and reset gate $r_{t}$. The outcome $h_{t}$ is defined by either present input $x_{t}$ or prior layer $h_{t-1}$ in the control of these 2 gates. The outcome of gates and the GRU unit can be computed as:

(14) $$r_{t}=\sigma (W_{r}x_{t}+U_{r}h_{t-1}+b_{r})\;z_{t}=\sigma \left(W_{z}x_{t}+U_{z}h_{t-1}+b_{z}\right)\;\;\tilde{h_{t}}=tanh[W_{h}x_{t}+U_{h}(r_{t}⊙h_{t-1})+b_{h}]\;h_{t}=(1-z_{t})⊙h_{t-1}+z_{t}⊙h_{t}$$

where $W_{r}$, $U_{r}$, $W_{z},$ $U_{z},$ $W_{h}$, and $U_{h}$ refer to the weighted matrices. $b_{r}$, $b_{z},$ and $b_{h}$ signifies the synthesis of bias vectors for input $x_{t}$ and preceding layer $h_{t-1},\;⊙$ stands for the Hadamard products, σ implies the logistic sigmoid function, and tanh describes the hyperbolic tangent activation function.

These methods with bidirectional design can learn data from preceding and subsequent data once controlled with the present data. The BiGRU technique is defined depending on the layer of 2 GRUs that are unidirectional in opposite directions. One GRU that moves forward starts with the beginning of the data order, and the other GRU that moves backward starts from the finish of the data order. This permits the data from either the future or past to influence the existing layers. The BiGRU is determined as:

(15) $$\vec{h_{t}}=GRU_{fwd}(x_{t},\;\vec{h_{t-1}})\;\overset{\leftarrow }{h_{t}}=GRU_{bwd}\left(x_{t},\;\overset{\leftarrow }{h_{t+1}}\right)\;\;h_{t}=\vec{h_{t}}⊕\overset{\leftarrow }{h_{t}}\;$$

In which, $\vec{h_{t}}$ refers to the layer of the forward GRU, $\overset{\leftarrow }{h_{t}}$ denotes the layer of the backward GRU, ⊕ and stands for the procedure of concatenating 2 vectors.

The MBGRU is an advanced category of the conventional GRU that increases its abilities by integrating the notion of multi-head attention. This model incorporates the strengths of attention mechanisms and bi-directional processing for capturing long-term reliance and considering significant information in sequential data. In MBGRU, the architecture has been established with numerous attention heads, permitting it to appear for promptly various sections of the input sequence. The bi-directional feature of the GRU allows the network to measure data from both previous and upcoming time stages, which enables a highly extensive understanding of temporal dependencies. The combination of multi-head attention also increases the model’s capability for capturing intricate patterns and interconnections within the data, which makes it appropriate for tasks, namely time series analysis, natural language processing (NLP), and other applications wherein contextual data can be vital. The MBGRU method represents a robust solution for tasks that need subtle analysis of sequential information by integrating the aids of multi-head attention mechanisms and bi-directionality.

## 4. Performance Validation

The proposed model is simulated using the Python 3.6.5 tool on PC i5-8600k, GeForce 1050 Ti 4 GB, 16 GB RAM, 250 GB SSD, and 1 TB HDD. The parameter settings are given as follows: learning rate: 0.01, dropout: 0.5, batch size: 5, epoch count: 50, and activation: ReLU.

**Figure 2:**
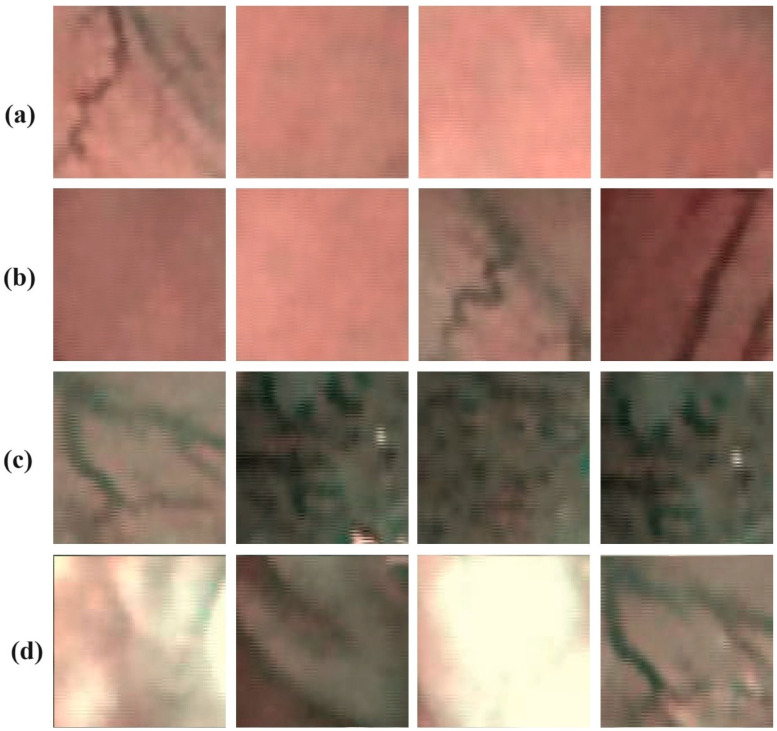
Sample images (**a**) Hbv; (**b**) He; (**c**) IPCL; (**d**) Le.

In this section, the LCA detection outcome of the ALCAD-DMODL technique is carried out on the throat region image dataset, which contains 1320 samples using four classes, as described in Table 1. Figure 2 signifies the sample images.

**Figure 3:**
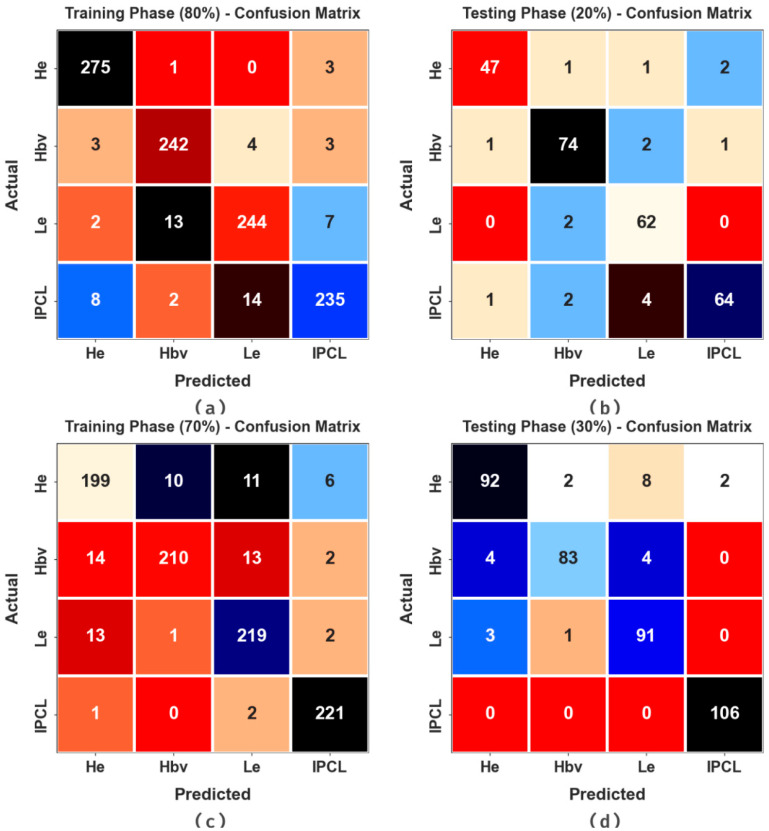
Confusion matrices of (**a**,**b**) TRPH/TSPH of 80:20 and (**c**,**d**) TRPH/TSPH of 70:30.

Figure 3 demonstrates the confusion matrices produced by the ALCAD-DMODL model below 80:20 and 70:30 of TRPH/TSPH. The results indicate the effectual detection and classification of all four classes.

**Figure 4:**
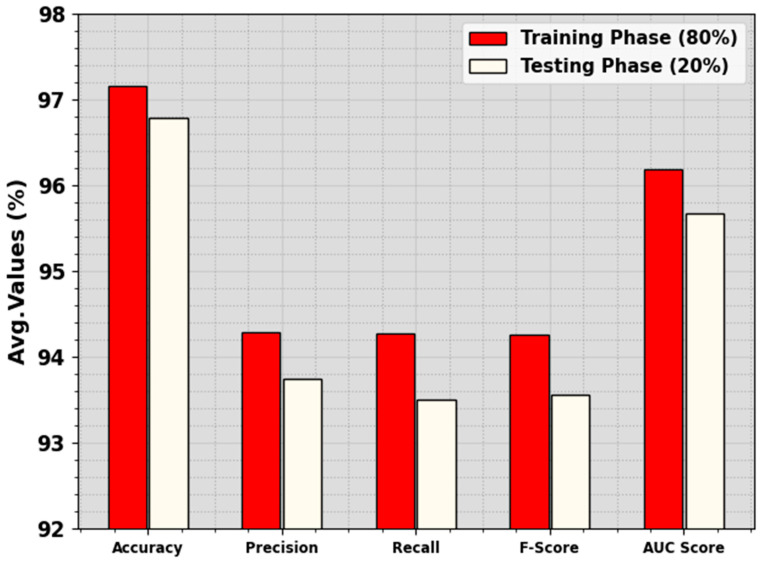
Average of ALCAD-DMODL technique under 80:20 of TRPH/TSPH.

In Table 2 and Figure 4, an overall LCA recognition outcome of the ALCAD-DMODL method under 80:20 of TRPH/TSPH is shown. The result inferred that the ALCAD-DMODL technique has effectual detection of all four classes. With 80% of TRPH, the ALCAD-DMODL model provides an average $accu_{y}$, $prec_{n}$, $reca_{l}$, $F_{score}$, and $AUC_{score}$ of 97.16%, 94.29%, 94.27%, 94.26%, and 96.19%, respectively. Furthermore, with 20% of TSPH, the ALCAD-DMODL approach delivers average $accu_{y}$, $prec_{n}$, $reca_{l}$, $F_{score}$, and $AUC_{score}$ of 96.78%, 93.74%, 93.51%, 93.56%, and 95.67%, correspondingly.

**Figure 5:**
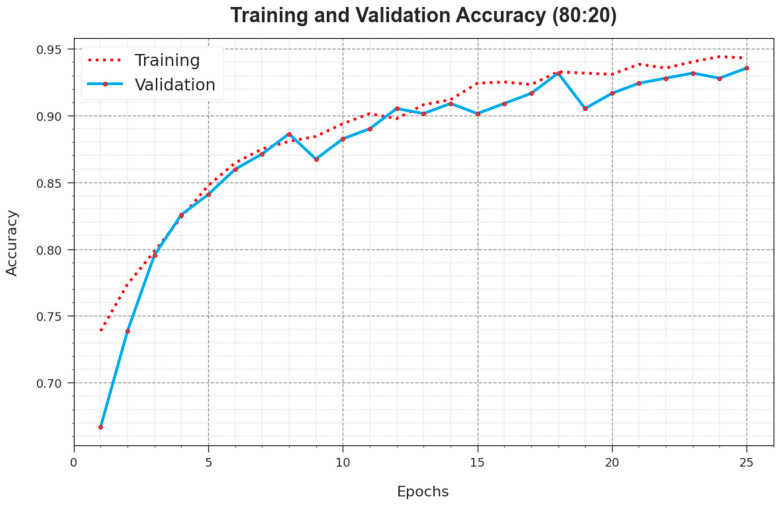
$Accu_{y}$ curve of ALCAD-DMODL technique under 80:20 of TRPH/TSPH.

The $accu_{y}$ curves for training (TR) and validation (VL) exposed in Figure 5 for the ALCAD-DMODL technique under 80:20 of TRPH/TSPH provide valuable insights into its performance below many epochs. Particularly, there is steady development in both TR and TS $accu_{y}$ to growing epochs, representing the model’s ability to learn and identify patterns from both TR and TS data. The upward trend in TS $accu_{y}$ underlines the model’s flexibility to the TR dataset and its capability to create precise forecasts on hidden data, emphasizing robust generalization skills.

**Figure 6:**
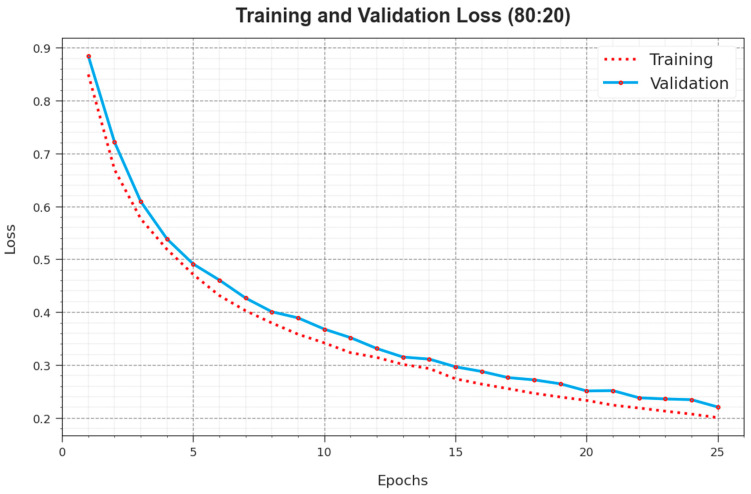
Loss curve of ALCAD-DMODL technique under 80:20 of TRPH/TSPH.

Figure 6 delivers a complete summary of TR and TS loss values for the ALCAD-DMODL technique under 80:20 of TRPH/TSPH through numerous epochs. The TR loss steadily drops as the model improves its weights to minimize classification errors on both datasets. The loss curves exemplify the model’s alignment with TR data, highlighting its aptitude to capture patterns efficiently in both datasets. Significant is the continuous alteration of parameters in the ALCAD-DMODL technique, marked by diminishing discrepancies amid predictions and actual TR labels.

**Figure 7:**
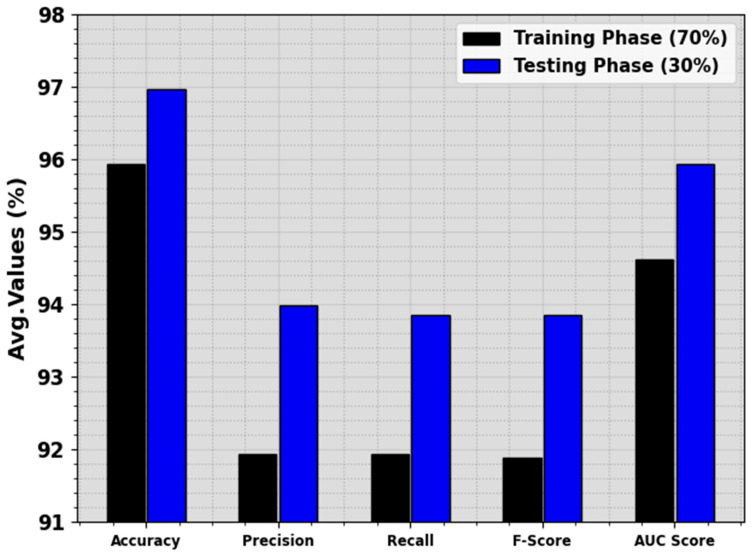
Average of ALCAD-DMODL technique under 70:30 of TRPH/TSPH.

In Table 3 and Figure 7, a complete LCA recognition outcome of the ALCAD-DMODL model below 70:30 of TRPH/TSPH is shown. The consequence inferred that the ALCAD-DMODL technique has effective detection of all four classes. With 70% of TRPH, the ALCAD-DMODL method provides average $accu_{y}$, $prec_{n}$, $reca_{l}$, $F_{score}$, and $AUC_{score}$ of 95.94%, 91.94%, 91.94%, 91.89%, and 94.62%, respectively. Furthermore, with 30% of TSPH, the ALCAD-DMODL technique offers average $accu_{y}$, $prec_{n}$, $reca_{l}$, $F_{score}$, and $AUC_{score}$ of 96.97%, 93.98%, 93.86%, 93.85%, and 95.93%, separately.

**Figure 8:**
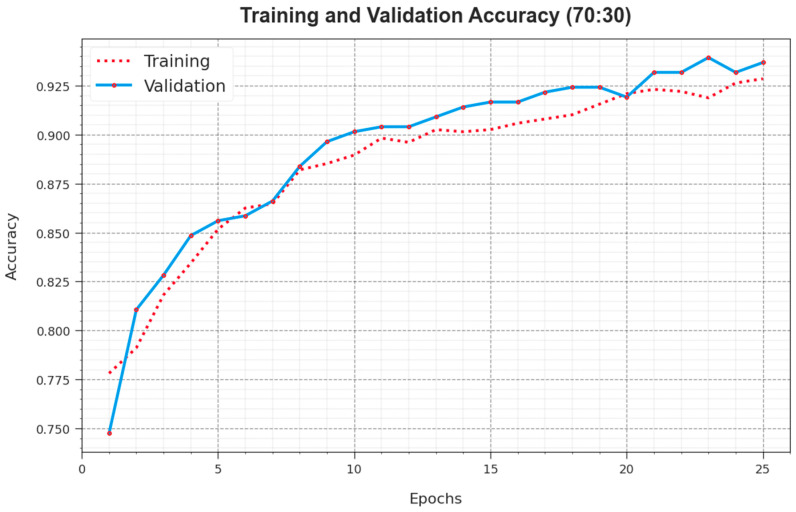
$Accu_{y}$ curve of ALCAD-DMODL technique under 70:30 of TRPH/TSPH.

The $accu_{y}$ curves for TR and VL presented in Figure 8 for the ALCAD-DMODL technique under 70:30 of TRPH/TSPH provide valuable insights into its performance below several epochs. Particularly, there is a reliable enhancement in both TR and TS $accu_{y}$ with collective epochs, demonstrating the model’s expertise in learning and diagnosing patterns from both TR and TS data. The upward trend in TS $accu_{y}$ underlines the model’s flexibility to the TR dataset and its capacity to create exact forecasts on unseen data, prominence robust generalization skills.

**Figure 9:**
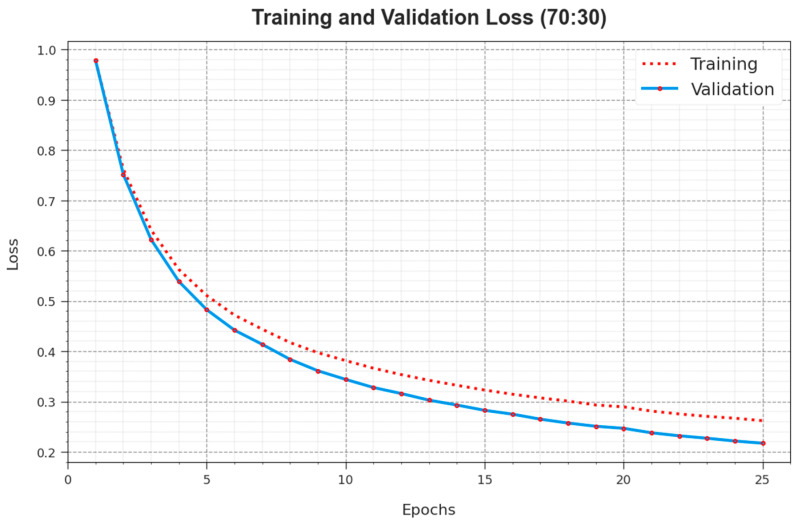
Loss curve of ALCAD-DMODL technique under 70:30 of TRPH/TSPH.

Figure 9 offers an inclusive overview of the TR and TS loss values for the ALCAD-DMODL model under 70:30 of TRPH/TSPH across numerous epochs. The TR loss dependably reduces as the method perfects its weights to decrease classification errors on both datasets. The loss curves clarify the model’s position with TR data, underscoring its capability to capture patterns well in both datasets. Noteworthy is the endless refinement of parameters in the ALCAD-DMODL model, intended to diminish discrepancies between predictions and actual TR labels.

**Figure 10:**
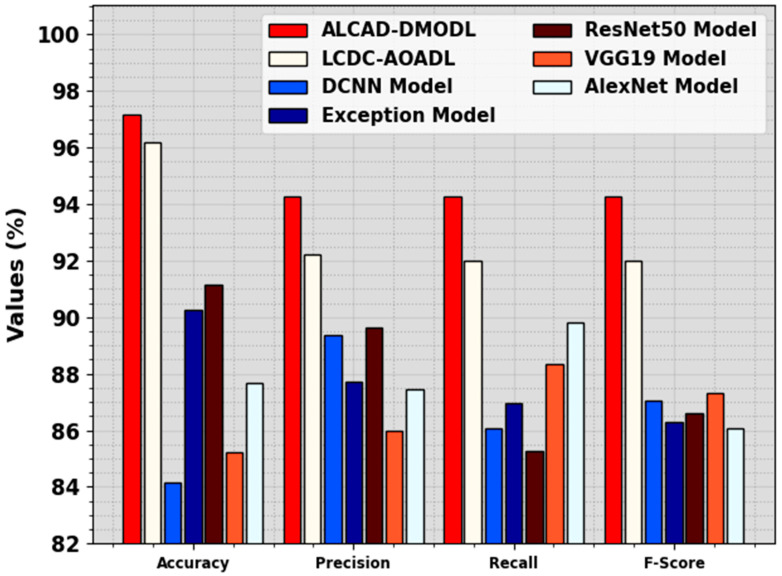
Comparative analysis of ALCAD-DMODL methodology with other models.

Table 4 and Figure 10 illustrate a comprehensive comparative analysis of ALCAD-DMODL methodology with other recent techniques [11]. The simulation values imply that the ALCAD-DMODL method has outperformed enhanced performances. Concerning $accu_{y}$, the ALCAD-DMODL technique has obtained a higher $accu_{y}$ of 97.16%. On the other hand, the LCDC-AOADL, DCNN, Exception, ResNet50, VGG19, and AlexNet approaches have achieved lesser $accu_{y}$ of 96.18%, 84.16%, 90.27%, 91.13%, 85.23%, and 87.66%, respectively. Additionally, based on $prec_{n}$, the ALCAD-DMODL methodology has attained a greater $prec_{n}$ of 94.29%. In addition, the LCDC-AOADL, DCNN, Exception, ResNet50, VGG19, and AlexNet techniques have succeeded lesser $prec_{n}$ of 92.24%, 89.37%, 87.72%, 89.62%, 85.98%, and 87.45%, correspondingly. Lastly, based on $F_{score}$, the ALCAD-DMODL methodology has gained a higher $F_{score}$ of 94.26%. On the other hand, the LCDC-AOADL, DCNN, Exception, ResNet50, VGG19, and AlexNet methods have reached a lesser $F_{score}$ of 91.99%, 87.06%, 86.27%, 86.61%, 87.30%, and 86.06%, individually.

**Figure 11:**
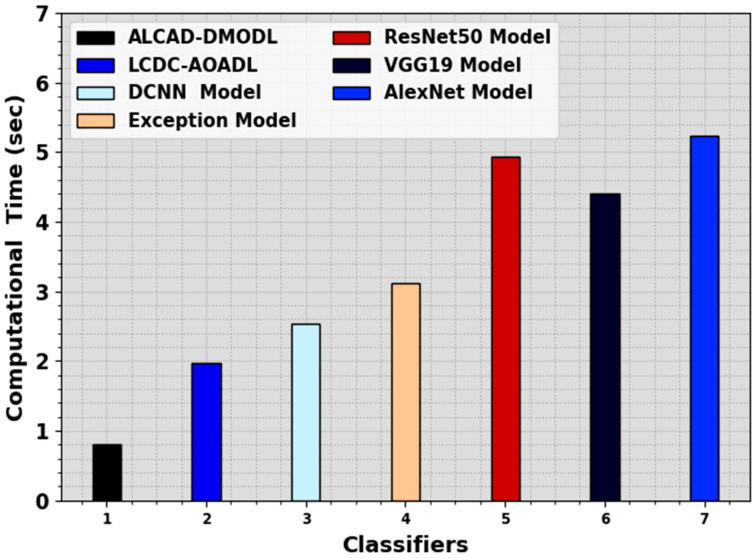
CT analysis of the ALCAD-DMODL system with other models.

In Table 5 and Figure 11, a complete computational time (CT) analysis of the ALCAD-DMODL technique with other existing models is displayed. The outcome values suggest that the ALCAD-DMODL model has outperformed superior performances. With esteem to CT, the ALCAD-DMODL method has gained a lesser CT of 0.80 s. On the other hand, the LCDC-AOADL, DCNN, Exception, ResNet50, VGG19, and AlexNet methodologies have reached lesser $accu_{y}$ of 1.98 s, 2.54 s, 3.12 s, 4.94 s, 4.41 s, and 5.24 s, individually. These results confirmed the enhanced performance of the LCA detection process.

## 5. Conclusions

In this study, we have presented an ALCAD-DMODL methodology. The main aim of the ALCAD-DMODL methodology is to recognize the existence of LCA using the DL model. The presented ALCAD-DMODL technique comprises MF-based preprocessing, an EfficientNet-B0-based feature extractor, DMO-based parameter tuning, and MBGRU-based classification. In addition, the ALCAD-DMODL technique involves the EfficientNet-B0 model for deriving feature vectors from the pre-processed images. For optimal hyperparameter tuning of the EfficientNet-B0 model, the DMO algorithm can be applied to select the parameters. Finally, the MBGRU model could be applied for the recognition and classification of LCA. The simulation result analysis of the ALCAD-DMODL method is carried out under the throat region image dataset. The comparison study stated the supremacy of the ALCAD-DMODL technique in terms of distinct measures.

## Figures and Tables

**Table 1:** Details on the database.

Name	Classes	No. of Instances
Healthy Tissue	He	330
Hypertrophic Blood Vessels	Hbv	330
Leukoplakia	Le	330
Abnormal IPCL-like Vessel	IPCL	330
Total No. of Instances		1320

**Table 2:** LCA detection outcome of ALCAD-DMODL technique under 80:20 of TRPH/TSPH.

Classes	$Accu_{y}$	$Prec_{n}$	$Reca_{l}$	$F_{Score}$	$AUC_{Score}$
TRPH (80%)					
He	98.39	95.49	98.57	97.00	98.45
Hbv	97.54	93.80	96.03	94.90	97.02
Le	96.21	93.13	91.73	92.42	94.73
IPCL	96.50	94.76	90.73	92.70	94.55
**Average**	**97.16**	**94.29**	**94.27**	**94.26**	**96.19**
**TSPH (20%)**					
He	97.73	95.92	92.16	94.00	95.61
Hbv	96.59	93.67	94.87	94.27	96.09
Le	96.59	89.86	96.88	93.23	96.69
IPCL	96.21	95.52	90.14	92.75	94.29
**Average**	**96.78**	**93.74**	**93.51**	**93.56**	**95.67**

**Table 3:** LCA detection outcome of ALCAD-DMODL technique under 70:30 of TRPH/TSPH.

Class Labels	$Accu_{y}$	$Prec_{n}$	$Reca_{l}$	$F_{Score}$	$AUC_{Score}$
**TRPH (70%)**					
He	94.05	87.67	88.05	87.86	92.02
Hbv	95.67	95.02	87.87	91.30	93.13
Le	95.45	89.39	93.19	91.25	94.71
IPCL	98.59	95.67	98.66	97.14	98.62
**Average**	**95.94**	**91.94**	**91.94**	**91.89**	**94.62**
**TSPH (30%)**					
He	95.20	92.93	88.46	90.64	93.03
Hbv	97.22	96.51	91.21	93.79	95.11
Le	95.96	88.35	95.79	91.92	95.90
IPCL	99.49	98.15	100.00	99.07	99.66
**Average**	**96.97**	**93.98**	**93.86**	**93.85**	**95.93**

**Table 4:** Comparative analysis of ALCAD-DMODL methodology with other models. [11].

Classifiers	$Accu_{y}$	$Prec_{n}$	$Reca_{l}$	$F_{Score}$
ALCAD-DMODL	97.16	94.29	94.27	94.26
LCDC-AOADL	96.18	92.24	91.99	91.99
DCNN	84.16	89.37	86.07	87.06
Exception	90.27	87.72	86.98	86.27
ResNet50	91.13	89.62	85.28	86.61
VGG19	85.23	85.98	88.33	87.30
AlexNet	87.66	87.45	89.83	86.06

**Table 5:** CT analysis of the ALCAD-DMODL approach with other models.

Classifiers	Computational Time (s)
ALCAD-DMODL	0.80
LCDC-AOADL	1.98
DCNN	2.54
Exception	3.12
ResNet50	4.94
VGG19	4.41
AlexNet	5.24

## Data Availability

The data presented in this study are available in this article.
